# Detecting Range Shrinking From Historical Amphibian Species Occurrences Under Influence of Human Impacts: A Case Study Using the Chinese Giant Salamander, *Andrias davidianus*

**DOI:** 10.1002/ece3.70595

**Published:** 2024-11-19

**Authors:** Siqing Li, Wenyu Dai, Zhenkang Wang, Zhaoning Wu, Jiechen Wang

**Affiliations:** ^1^ Jiangsu Provincial Key Laboratory of Geographic Information Science and Technology, Key Laboratory for Land Satellite Remote Sensing Applications of Ministry of Natural Resources, School of Geography and Ocean Science Nanjing University Nanjing China; ^2^ Jiangsu Center for Collaborative Innovation in Geographical Information Resource Development and Application Nanjing China

**Keywords:** amphibians, climate change, habitat range loss, historical archives, human disturbance

## Abstract

Amphibian declines, driven by climate change (e.g., shifting temperatures, altered precipitation) and human activities like deforestation, agriculture, and urbanization, may lead to local extinctions. Quantifying the relative impact of climate change versus human influence remains challenging. This study uses species distribution models (SDMs) and nearly 1000 years of historical distribution data from ancient texts and local archives to reconstruct the past distribution range of the Chinese giant salamander ( *Andrias davidianus* ) and to assess the spatiotemporal shifts in its range over time. The results reveal that over the past millennium, the potential distribution range of the Chinese giant salamander consistently contracted, decreasing by 10% from the Ming Dynasty (1368–1644) to the Qing Dynasty (1644–1912) and a further 30% from the Qing Dynasty to the modern era. Losses are concentrated in eastern plains with abundant water bodies, resulting in available habitat reduction to 27% of the Qing Dynasty's area. Climate factors have been key in shaping the salamander's distribution, but our findings reveal that population density has consistently impacted its range throughout history, highlighting the lasting influence of human activity. Climate models project a about 10% decrease in suitable habitat by around 2090, with northward shifts in suitable habitat. Given the urgent threat of habitat loss and environmental degradation, immediate and effective actions are crucial to prevent the local extinction of the Chinese giant salamander, including habitat protection, environmental restoration, and strict regulations against hunting and habitat destruction. This study, analyzing the Chinese giant salamander's suitable habitat historically, identifies human activities as a pivotal force in early amphibian species decline in China, contributing valuable perspectives to future amphibian conservation and management.

## Graphical abstract

The Chinese giant salamander ( *Andrias davidianus* ) has experienced a significant contraction in its potential distribution range over the past millennium, primarily due to climate change and human activities. Our species distribution models reveal a 10% decline from the Ming Dynasty to the Qing Dynasty and a further 30% reduction into the modern era, with projections indicating a 10%–20% decrease in suitable habitat by 2090. Immediate conservation actions are necessary to prevent extirpation, focusing on habitat protection, environmental restoration, and strict regulations against hunting and habitat destruction.

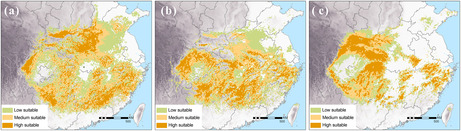

## 1 Introduction

Human activities, such as modern land use and other anthropogenic factors, have greatly accelerated habitat loss, driving widespread declines and extinctions in plant and animal populations (Di Marco and Santini 2015; Pimm et al. 2014), particularly amphibians (Stuart et al. 2004), over the past few centuries. Extinction events are typically protracted processes characterized by prolonged declines in species' geographic ranges and population sizes (Pimm and Raven 2000). The extinction of a species following ecological disturbances is not always immediate; it may unfold over decades, centuries, or even longer (Tilman et al. 1994). Therefore, studying contemporary populations of threatened species often provides limited information about the earlier dynamics leading to their current declines.

There is a growing recognition of the need to integrate historical datasets into conservation research and environmental management to formulate more comprehensive decision frameworks. (Grace et al. 2019; Li et al. 2015; Turvey, Crees, and Di Fonzo 2015; Zhang et al. 2018). This integration can provide unique insights into the long‐term extinction dynamics of species and ecosystems, which are beyond the scope of short‐term ecological studies (Turvey, Crees, and Di Fonzo 2015). Compared to geological and centennial‐scale studies, quantitative research on millennial‐scale impacts of climate change and human influences on species range changes remains limited due to the often fragmented or missing knowledge of historical species distributions (Bonebrake et al. 2010). In this context, the historical patterns of amphibians hold broader significance for understanding the extinction dynamics caused by human activities.

China, with its vast geographic area spanning about 9.6 million square kilometers and diverse ecosystems, including high mountains, plains, wetlands, forests, and coastal areas, is one of the world's most amphibian‐diverse countries (Xie et al. 2007). China also holds rich late Quaternary paleobiological and zooarchaeological records, including substantial data on amphibians (Jia and Gao 2016), which can provide important insights into the historical trends of regional biodiversity. Over the millennia, historical records in China have documented rare and large animals due to their economic and utilitarian value or conflicts with humans (Wen 2018). These records have been comprehensively studied within a quantitative analytical framework (Turvey et al. 2018; Wan et al. 2019; Zhao et al. 2023, 2018), but research on amphibian species has been limited.

The Chinese giant salamander ( *Andrias davidianus* ), the world's largest amphibian, is endemic to China and is classified as critically endangered by the International Union for Conservation of Nature (IUCN) Red List due to threats such as habitat loss and overexploitation (IUCN 2022). Its high evolutionary distinctiveness (Isaac et al. 2007) and extinction risk make it a top conservation priority (Turvey et al. 2018; Wang et al. 2004; Zhang et al. 2020). Once widespread across much of China, this species has experienced a significant decline due to habitat destruction and increased demand for its meat (Pan et al. 2016; Yan et al. 2018). Studies have assessed its current status and responses to both climate change and human activities, revealing that rising human population density, more than climate change, has caused substantial range loss and fragmentation (Chen et al. 2018; Wang et al. 2004; Zhang et al. 2020; Zhang et al. 2020). Historically, China's high population density and large‐scale conversion of forest land for agriculture have led to extensive deforestation, terracing, and other forms of land modification (Ge et al. 2008; Klein Goldewijk et al. 2011). Despite these known impacts, few studies have analyzed historical distribution data to examine how human activities have influenced amphibian species' distribution ranges across different periods. Based on this context, we hypothesize that human activities have been influencing the distribution of the Chinese giant salamander for several centuries. As population density has increased, the species' potential habitat has continuously contracted, particularly in regions with intensive land use and high population density.

This study, using the Chinese giant salamander as a case study, relies on historical distribution data various sources. It aims to: (1) estimate the habitat suitability distribution changes of the Chinese giant salamander over the past 1000 years and into the future (2030s, 2050s, 2070s, 2090s); (2) quantify the impact of bioclimatic, topographical, and human activity factors on the species' distribution range shifts. The research findings will improve our understanding of the processes and mechanisms of the salamander's response to human influence, providing a theoretical basis and guidance for conservation management in the region.

## 2 Materials and Methods

### 2.1 Species Distribution Records

*Andrias davidianus* is recognized as a species complex, recently divided into several species: *A. sligoi* (Turvey et al. 2019), *A. jiangxiensis* (Chai et al. 2022), and *A. cheni* (Gong et al. 2023). However, these classifications are considered preliminary, as further research on reproductive isolation is needed to confirm them as distinct species (Luo et al. 2023). Given the limitations of species occurrence data, which lack the morphological and genetic details required to differentiate these taxa, we chose to treat them collectively as *Andrias davidianus* . This approach offers a comprehensive perspective on the distribution of the Chinese giant salamander across China.

**FIGURE 1:**
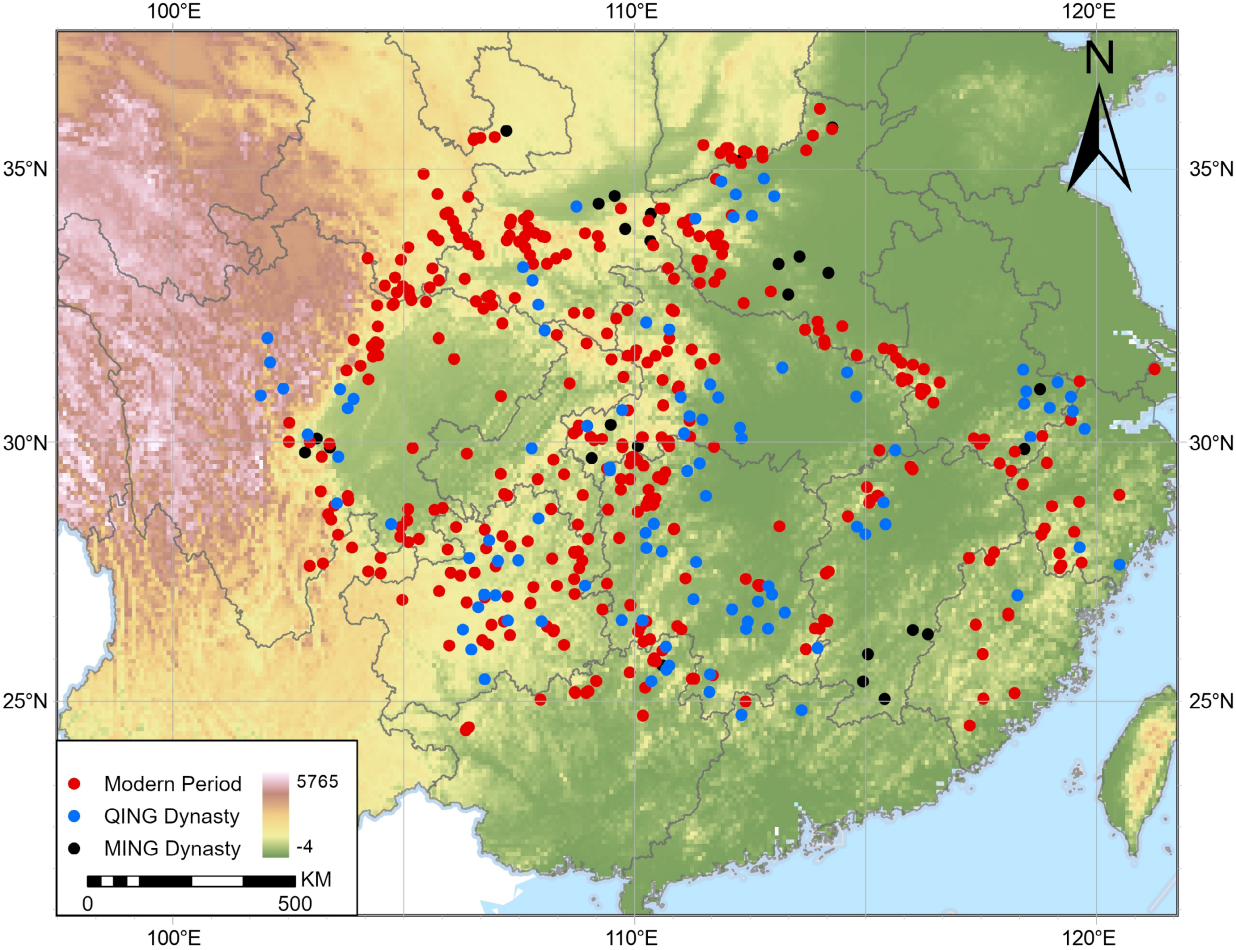
Map of historical and modern occurrence records of the Chinese giant salamander across mainland China, with the background layer representing elevation (m).

Historical distribution data for the Chinese giant salamander were sourced from Wen's comprehensive compendium (Wen 2018), which applied a rigorous approach to record compilation, including only those records supported by verifiable and credible evidence. To further enhance the accuracy and relevance of our analysis, we refined the dataset by selecting occurrence records that were precisely geolocated to the county level or specific mountain ranges and were accompanied by clearly defined temporal information (Data S1, Tables S1–S3). In total, 139 historical records (depicted as black and blue dots in Figure 1) from Wen's study were used, including 36 records from the Ming Dynasty (1368–1644) and 103 records from the Qing Dynasty (1644–1912).

The modern occurrence records (depicted as red dots in Figure 1) (*n* = 371), were derived from three primary sources: (1) 43 records post‐1911 from Wen's (2018) research; (2) six records post‐1911 from the Global Biodiversity Information Facility (GBIF.Org 2023) (Accessed 09/06/2020, Table S4); and (3) 322 records from Mi et al. (2022), who compiled data on the occurrence locations of Chinese amphibian species from literature since 1980.

To improve the reliability and accuracy of species distribution data, we employed a two‐step filtering process. First, the R package “spThin” (Aiello‐Lammens et al. 2015) was used to eliminate duplicate data points and reduce spatial autocorrelation. Then, ENMTools (Warren, Glor, and Turelli 2010) was applied to minimize spatial sampling bias by retaining only one occurrence point per 10 km × 10 km environmental data raster. This ensured each grid cell contained a single, accurate distribution point, enhancing the model's robustness. After filtering, 113 historical and 331 contemporary distribution points were used to train the models separately (Figure 1).

### 2.2 Environmental Predictors

#### 2.2.1 Bioclimatic Data

We sourced 19 standard bioclimatic variables and elevation data at a 10 km resolution from WorldClim 2.1 (Fick and Hijmans 2017). To minimize collinearity, we selected isothermality (bio3), minimum temperature of the coldest month (bio6), annual precipitation (bio12), and precipitation of the driest month (bio14) based on pairwise Pearson correlation coefficients (|*r*| < 0.70) (Zhou et al. 2009).

#### 2.2.2 Digital Elevation and Geomorphology Data

The digital elevation model (DEM) data used in this study were obtained from the National Earth System Science Data Center (https://www.geodata.cn), with a spatial resolution of 1 km. Geomorphological data, categorized into seven types (plains, plateaus, hills, low‐relief mountains, mid‐relief mountains, high‐relief mountains, and extensive high‐relief mountains), were derived from the Data Center for Resources and Environmental Sciences, Chinese Academy of Sciences (http://www.resdc.cn), as classified in the research by Zhou et al. (2009).

#### 2.2.3 Historical Human Activity Maps

We utilized maps related to human activities during the Ming and Qing dynasties provided by HYDE 3.2 dataset (Klein Goldewijk et al. 2017), which provides a temporal sequence of population and land use categories from 10,000 BCE to 2019 CE. The land use categories—urban, villages, croplands, pastures, seminatural, and wild—were generally allocated based on factors such as population density, soil suitability, slope, and proximity to water sources. Additionally, these allocations were guided by data from various yearbooks published by the Food and Agriculture Organization (FAO), providing a broad framework for understanding historical land use patterns. We specifically used the data for the year 1400 to represent the Ming Dynasty, 1700 to represent the Qing Dynasty, and 2010 for the modern era. For each of these periods, we conducted statistical analyses of population density and the proportions of different land use types.

#### 2.2.4 Climate Data

We considered four specific combinations of shared socioeconomic pathways (SSP) and representative concentration pathways (RCP) to simulate potential future scenarios: SSP5‐8.5, SSP3‐7.0, SSP2‐4.5, and SSP1‐2.6. These scenarios represent a range of future forcing pathways from high to low (Meinshausen et al. 2020; Riahi et al. 2017). By the end of the 21st century, global temperatures are projected to rise by 1.18°C per 100 years under SSP1‐2.6, 3.22°C per 100 years under SSP2‐4.5, 5.50°C per 100 years under SSP3‐7.0, and 7.20°C per 100 years under SSP5‐8.5 (Fan et al. 2020). To account for variability and enhance the robustness of our projections, we utilized CMIP6 climate projections generated by 10 global circulation models (GCMs): UKESM1‐0‐LL (Tang et al. 2023), MRI‐ESM2‐0 (Yukimoto et al. 2019), MIROC6 (Tatebe et al. 2019), IPSL‐CM6A‐LR (Boucher et al. 2020), INM‐CM5‐0 (Volodin et al. 2019), GISS‐E2‐1‐G (Kelley et al. 2020), EC‐Earth3‐Veg (EC‐Earth Consortium (EC‐Earth) 2019), CMCC‐ESM2 (Lovato et al. 2022), ACCESS‐CM2 (Bi et al. 2020), and MPI‐ESM1‐2‐HR (Müller et al. 2018). Using these projections, we assessed climate suitability estimates for the Chinese giant salamander under four SSP scenarios (SSP5‐8.5, SSP3‐7.0, SSP2‐4.5, and SSP1‐2.6) across four time periods: 2030s, 2050s, 2070s, and 2090s. This approach produced 160 potential distribution maps of the Chinese giant salamander (4 scenarios × 4 time periods × 10 GCMs).

In modeling the habitats of the Chinese giant salamander for both historical and modern periods, we used all the aforementioned data. However, to emphasize the impact of climate, future habitat modeling was based solely on climatic data, DEM data, and geomorphology type data, without incorporating future land use changes. The DEM and geomorphology data utilized in the modeling are sourced from current datasets, based on the expectation that significant changes to these variables will be minimal over the next 100 years.

### 2.3 Habitat Suitability Model

To model the distribution of the giant salamander, we employed an ensemble modeling approach using the biomod2 package in R (Thuiller et al. 2012), which integrates multiple algorithms to enhance predictive accuracy and robustness.

2.3.1

Seven algorithms available in biomod2 were selected to construct the ensemble models: Generalized Boosted Models (GBM), Classification Tree Analysis (CTA), Artificial Neural Networks (ANN), Surface Range Envelope (SRE), Flexible Discriminant Analysis (FDA), Random Forest (RF), and Maxent. A total of 10,000 pseudo‐absence data points were generated, with each model run replicated 10 times. Each replicate utilized a distinct set of pseudo‐absences, and the final ensemble predictions were obtained by averaging these replicates. The dataset was partitioned with 80% of the data used for model training and 20% reserved for validation. The ensemble models were constructed using a full consensus approach, where the contribution of each algorithm was weighted based on a true skill statistic (TSS) threshold of > 0.7, ensuring that only high‐performing models contributed to the final predictions.

In addition, we employed the maximum entropy model (Phillips, Anderson, and Schapire 2006), which is particularly useful for its ability to model species distributions without relying on pseudo‐absence data. The Maxent model was optimized and run using a range of parameter settings within the ENMeval R package (Muscarella et al. 2014). The results from the Maxent model, presented in Data S2, offer a comparative perspective that further validates the robustness of the ensemble models.

### 2.4 Model Evaluation

#### 2.4.1 Performance Metrics

To assess the overall accuracy of ensemble models, we employed metrics from the receiver operating characteristic (ROC) curve and the TSS. We calculated the area under the curve (AUC) of the ROC (Metz 1978; Phillips, Anderson, and Schapire 2006) and subsequently transformed this AUC into a Partial‐ROC (Townsend Peterson, Papeş, and Eaton 2007). The calculation of TSS is based on the sum of sensitivity (the proportion of presences correctly predicted) and specificity (the proportion of absences correctly predicted) minus one. TSS values greater than 0.6 indicate good predictive performance, values between 0.2 and 0.6 suggest fair to moderate performance, and values below 0.2 are considered poor (Landis and Koch 1977).

#### 2.4.2 Similarity Analysis

To assess the consistency between model outputs, we calculated the Spearman correlation coefficient by comparing the habitat suitability maps generated by the MaxEnt model and the ensemble model for each time period (Ming, Qing, and modern).

### 2.5 Variable Importance

Jackknife testing was conducted to assess the contribution and importance of each environmental variable in predicting the species' potential distribution (Yang et al. 2013). This technique systematically removes one variable at a time to assess its influence on model performance, identifying the key factors that drive species distribution. By analyzing the impact of excluding individual variables, this method highlights those with the most significant contributions to habitat suitability.

Species response curves were generated to explore the relationship between habitat suitability and environmental factors, offering insights into the species' ecological tolerance and habitat preferences. The x‐axis represents the range of individual environmental variable values, while the y‐axis shows the predicted probability of favorable conditions generated by the Maxent model. Positive trends indicate a favorable response to the variable, while negative trends suggest limiting conditions. The diversity in response curve patterns helps pinpoint optimal environmental thresholds necessary for species persistence (Akyol, Örücü, and Arslan 2020).

### 2.6 Species Range Change

To track changes in species distribution over time, we applied the maximum training sensitivity plus specificity (MTSS) threshold to transform the probability outputs into clear presence/absence binary maps, ensuring a precise representation of species distribution dynamics (Liu et al. 2005). This threshold optimally balances sensitivity (true positive rate) and specificity (true negative rate), effectively managing errors of omission and commission based on the training data's error matrix.

To predict distribution under various future scenarios, we utilized the binary outputs from 10 GCMs across each period (total of four periods) and SSP (four types). We applied a majority voting rule, where a grid cell is considered suitable if 5 or more of the 10 GCMs predict presence. Using these maps, we determined the potential suitable habitat under future climate scenarios by calculating the number of grid cells with a value of 1 (presence) and 0 (absence). We also compared the current binary maps with the future binary maps to create change maps. This approach allowed us to estimate the persistence, loss, gain, and emergence of potentially suitable habitats.

#### 2.6.1 Water Body Distribution Analysis

From the Ming dynasty to the modern era, the vanished potential habitat areas are mostly located in the eastern plains, characterized by abundant water bodies such as ponds and streams, which are the actual habitats of the Chinese giant salamander. After modeling the species distribution for different periods, we analyzed the water body coverage within each distribution range. The water body distribution data were sourced from the “Environmental Sustainability Indicators Compilation” by Columbia University (CIESIN 2018). These data include various water bodies such as ponds and streams. We calculated the water body coverage in the potential distribution areas during the Qing dynasty and the modern era to assess changes over time. This analysis helped in understanding the habitat characteristics and their changes, thereby contributing to the species' current distribution trends.

## 3 Results

### 3.1 Model Optimization

Using ensemble modeling through the biomod2 package, we modeled the spatial distribution of the Chinese giant salamander for the Ming, Qing, and modern periods. The performance metrics for these models demonstrated strong predictive accuracy. The AUC values ranged from 0.961 to 0.968, and the TSS values varied from 0.830 to 0.841, across all three periods (Table 1). These outcomes underscore the effectiveness and reliability of the ensemble models in representing the ecological niche of the Chinese giant salamander across different historical and contemporary periods.

**TABLE 1:** Performance metrics (AUC and TSS) of Biomod2 ensemble models for habitat suitability of Chinese giant salamander across different historical periods.

Period	AUC	TSS
Ming	0.968	0.841
Qing	0.961	0.833
Modern	0.963	0.830

The results from the MaxEnt model were also analyzed for comparison and are provided in Data S2. The high correlation between the predictions of the MaxEnt model and the ensemble models (0.948 for Ming, 0.952 for Qing, and 0.938 for Modern) further supports the robustness of the ensemble modeling approach.

### 3.2 Species Distribution Shifts

The ensemble model results (Table 2) showed a suitable habitat area of 1.40 × 10^12^ km^2^ during the Ming Dynasty, decreasing by 9.46% to 1.27 × 10^12^ km^2^ in the Qing Dynasty, and further reducing by 27.38% to 9.23 × 10^11^ km^2^ in the Modern period. The centroid shifted from 112.44° E, 30.23° N in the Ming Dynasty to 110.49° E, 30.20° N in the Modern period. The average elevation of suitable habitats also increased from 441.92 m in the Ming Dynasty to 864.59 m in the Modern period.

**TABLE 2:** Species distribution shifts for the Chinese giant salamander across different historical periods.

Time period	Suitable area (km^2^)	Reduction ratio (%)	Average elevation (m)	Waterbody area (km^2^)	Centroid (longitude, latitude)
Ming dynasty	1.40 × 10^12^	—	442	1.48 × 10^4^	112.44° E, 30.23° N
Qing dynasty	1.27 × 10^12^	9.46	458	1.47 × 10^4^	112.13° E, 29.67° N
Modern	9.23 × 10^11^	27.38	865	0.32 × 10^4^	110.49° E, 30.20° N

Additionally, the analysis include the water area within the suitable habitats. The suitable water area during the Qing Dynasty was 1.47 × 10^4^ km^2^, which significantly decreased to 3.24 × 10^3^ km^2^ in the Modern period, representing a reduction of approximately 78%.

**FIGURE 2:**
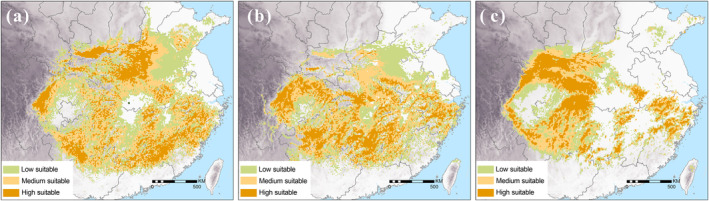
Potential suitable habitat of the Chinese giant salamander in China during (a) the Ming Dynasty, (b) the Qing Dynasty, and (c) the Modern period based on ensemble model results. The background represents elevation displayed in grayscale. Colors indicate habitat suitability: Low (green), medium (yellow), and high (orange).

In Figure 2, we present the potential suitable habitat of the Chinese giant salamander during the Ming Dynasty, Qing Dynasty, and Modern period based on ensemble model results. The results from the MaxEnt model, which are consistent with these findings, are provided in Data S2.

We analyzed human population density and the proportions of different land use types across these time periods (Table 3). Over time, the distribution range of the Chinese giant salamander showed a declining trend, while human population density increased rapidly, especially in the modern era. The proportions of urban and village areas—characterized by concentrated human populations—expanded, whereas the share of seminatural and wild areas, with lower levels of human alteration, diminished.

**TABLE 3:** Human population density and land use distribution in China during the historical periods.

Period	Human population density (people/km^2^)	Urban and village (%)	Seminatural and wild (%)
1400	7	1.1	64.8
1700	12	1.6	62.1
2010	142	48.34	18.5

### 3.3 Projected Distribution

**FIGURE 3:**
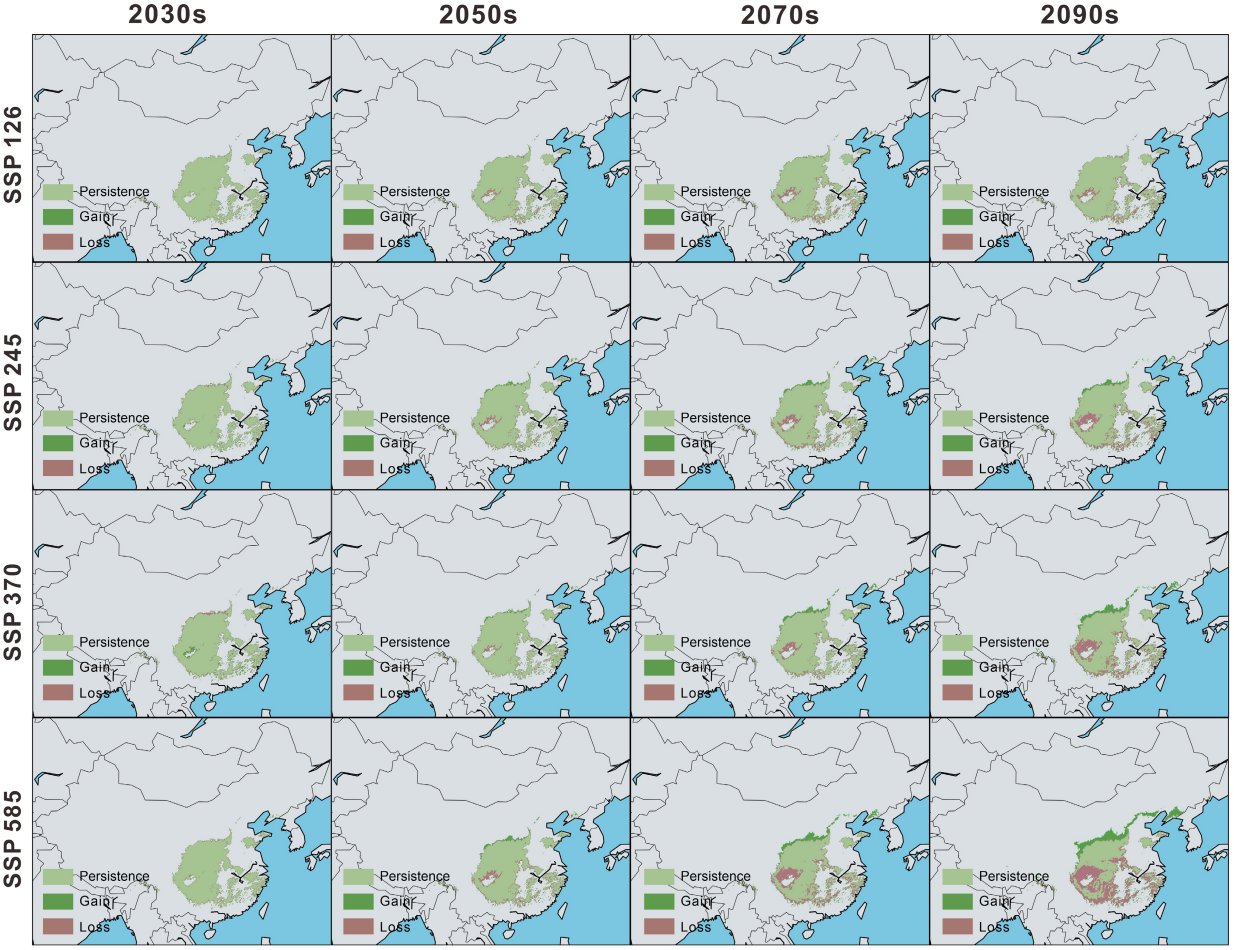
Projected changes in suitable habitat for the Chinese giant salamander under four SSP scenarios (SSP1‐2.6, SSP2‐4.5, SSP3‐7.0, SSP5‐8.5) across four time periods (2030s, 2050s, 2070s, 2090s). The maps illustrate shifts in habitat suitability, derived from ensemble model predictions. Light green areas represent persistence of suitable habitat, where conditions remain favorable over time. Reddish‐brown areas indicate habitat loss, where current suitable habitats are expected to become unsuitable. Dark green areas denote habitat gain, reflecting regions where new suitable habitats are projected to emerge.

The projected distribution of the Chinese giant salamander under various SSP scenarios and future time periods is illustrated in Figure 3. The maps display the potential suitable habitats changes for the 2030s, 2050s, 2070s, and 2090s under the SSP126, SSP245, SSP370, and SSP585 scenarios.

The distribution patterns vary across the different SSP scenarios and time periods. Under SSP126, the suitable habitat remains relatively stable from the 2030s to the 2090s, with minor reductions in the 2090s. In contrast, SSP245 shows a gradual decrease in suitable habitat over time, particularly noticeable by the 2090s. SSP370 and SSP585 scenarios both exhibit an initial increase in suitable habitat in the 2030s and 2050s, followed by a decline in the later decades, with SSP585 showing the most significant reduction by the 2090s.

The quantitative analysis of suitable habitat areas is summarized in Table 4. Under the SSP126 scenario, suitable habitat fluctuates modestly, with a 3.9% increase in the 2050s followed by a slight 4.5% decline by the 2090s. In the SSP245 scenario, suitable habitat shows a consistent reduction, with a total decrease of 6.5% by the 2090s. For SSP370, there is an initial 2.8% decline by the 2050s, followed by a further reduction of 8.9% by the 2090s. Similarly, the SSP585 scenario reaches a peak in the 2030s but then decreases by 11.6% by the end of the century.

**TABLE 4:** Projected suitable habitat area for Chinese giant salamander under various SSP scenarios and time periods (km^2^).

	2030s	2050s	2070s	2090s
SSP126	1.28 × 10^12^	1.33 × 10^12^	1.28 × 10^12^	1.27 × 10^12^
SSP245	1.39 × 10^12^	1.35 × 10^12^	1.31 × 10^12^	1.30 × 10^12^
SSP370	1.45 × 10^12^	1.41 × 10^12^	1.40 × 10^12^	1.32 × 10^12^
SSP585	1.38 × 10^12^	1.34 × 10^12^	1.28 × 10^12^	1.22 × 10^12^

**FIGURE 4:**
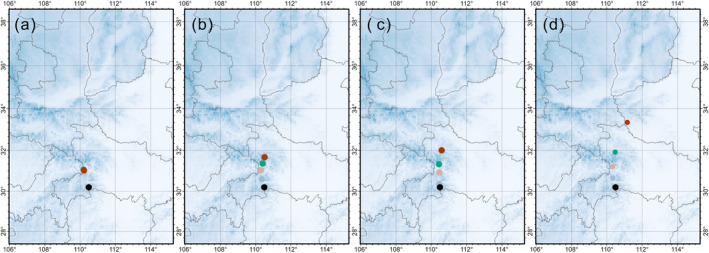
Centroid movement of future species distribution model (SDM) projections for the giant salamander under different scenarios of climate change. Scenario (a) SSP126 depicts an increase in temperature of 1.8°C, (b) SSP245 with an increase of 3.2°C, (c) SSP370 with an increase of 5.5°C, and (d) SSP585 with an increase of 7.2°C by the end of the 21st century. The maps are displayed with the DEM as the background.

Figure 4 illustrates the centroid movement of future binary species distribution model (SDM) projections under the SSP126, SSP245, SSP370, and SSP585 scenarios, with DEM as the background. The movement of the centroids represents the shift in the geographical center of suitable habitats over time. This analysis helps in understanding the potential migratory patterns of the Chinese giant salamander in response to changing climatic conditions.

These results suggest that the future distribution of the Chinese giant salamander is highly sensitive to climate change, with the extent of suitable habitats expected to vary significantly under different emission scenarios. The SSP370 scenario appears to provide the most favorable conditions for the species in the mid‐21st century, while SSP585 predicts the most substantial habitat loss by the century's end. The centroid movement analysis further indicates a northward shift in suitable habitats.

### 3.4 Importance of Environmental Factors

Across the Ming Dynasty, Qing Dynasty, and the modern era, the three factors contributing the most to the distribution of the Chinese giant salamander are consistently minimum temperature of the coldest month (bio6), precipitation of the driest month (bio14), and human population density (Table 5). The cumulative contribution rates of these variables were 78.7% in the Ming Dynasty, 80.4% in the Qing Dynasty, and 58.4% in the modern era. Notably, human population density emerged as the most significant influencing factor during the Ming and Qing Dynasties, with contribution rates reaching 35% and 50.1%, respectively. However, in the modern era, the importance of human population density has decreased, possibly due to population density nearing saturation, and the distribution of the giant salamander being more constrained by other environmental factors.

**TABLE 5:** Percent contribution and permutation importance of environmental variables to model the distribution of Chinese giant salamander across distinct time intervals.

Variable	Ming Dynasty (1368–1644)		Qing Dynasty (1644–1911)		Modern period (after 1911)	
	Percent contribution	Permutation importance	Percent contribution	Permutation importance	Percent contribution	Permutation importance
Isothermality	7.5	4.6	3.3	4.3	14.1	14.7
Minimum temperature of the coldest month	33	80.3	22.1	62	21	69.5
Annual precipitation	0.4	0.5	3.7	0.9	3.9	1.6
Precipitation of the driest month	10.7	0	8.2	3.1	22.1	5.2
DEM	7.1	8.6	5.4	6.2	12.2	5.5
Geomorphology type	6.3	5	7.1	6.8	6.4	0.1
Land‐use type	0.1	0	0.2	0	5	1.6
Human population density	35	1	50.1	7.8	15.3	1.9

**FIGURE 5:**
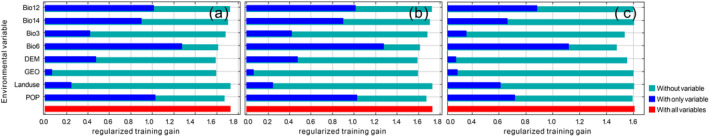
Jackknife analysis of environmental variable importance in modeling the distribution of the Chinese giant salamander ( *Andrias davidianus* ) across three historical periods: (a) Ming Dynasty, (b) Qing Dynasty, and (c) Modern Period. The y‐axis lists the environmental variables: Bio12 (annual precipitation), Bio14 (precipitation of the driest month), Bio3 (isothermality), Bio6 (minimum temperature of the coldest month), DEM, GEO (geomorphology type), Landuse (land use type), and POP (human population density).

From the perspective of variable importance ranking, minimum temperature of the coldest month (bio6) consistently exhibited high importance throughout the studied periods. Results from the Jackknife test of variable importance further indicate that starting from the Ming Dynasty, annual precipitation (bio12), minimum temperature of the coldest month (bio6), and human population density have the greatest impact on predicting the distribution of the giant salamander when compared to other factors estimated by Jackknife tests (Figure 5).

**FIGURE 6:**
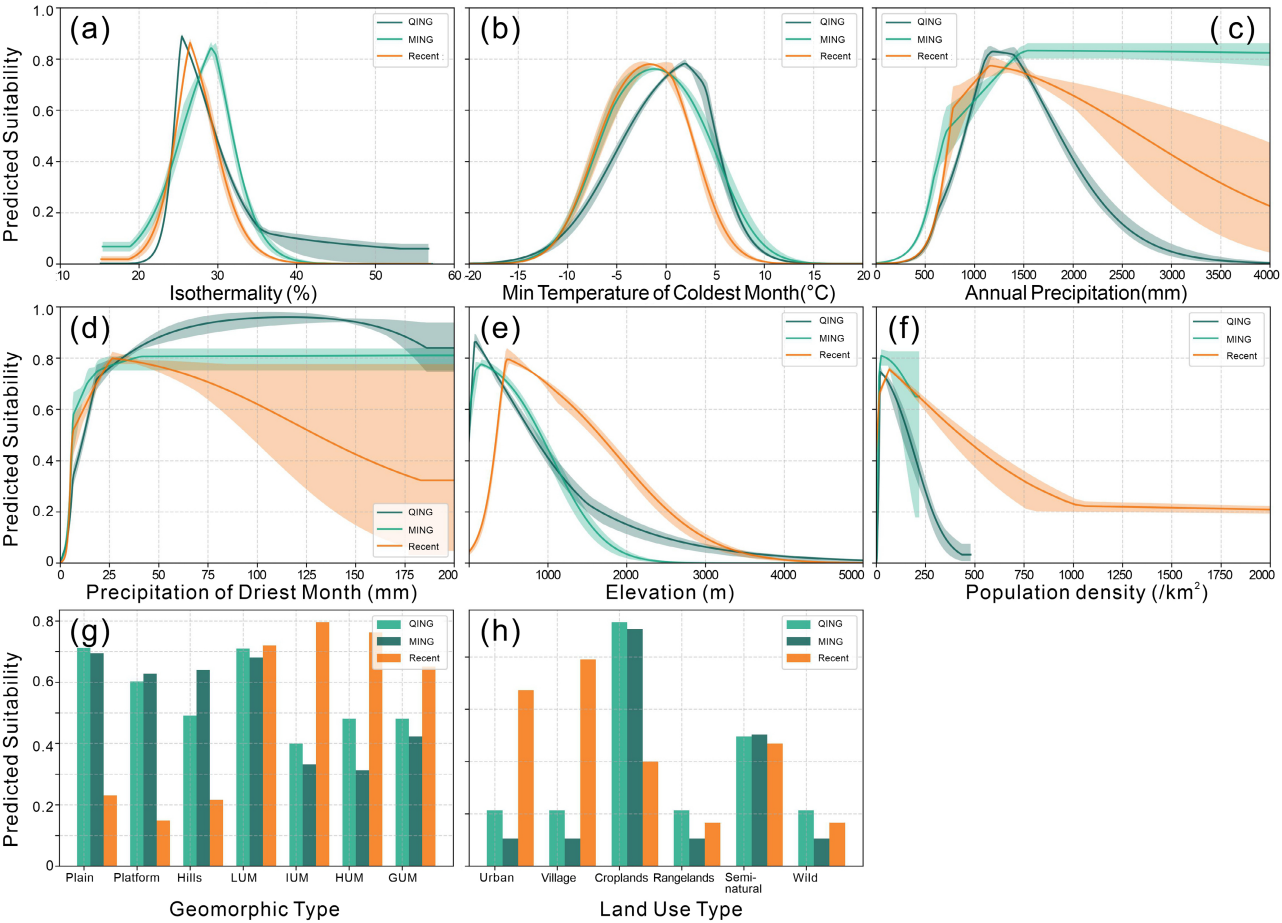
Species response curves for the Chinese giant salamander in the Ming Dynasty, Qing Dynasty, and modern times depict the relationship between the presence probability and (a) Isothermality, (b) Min temperature of coldest month, (c) Annual precipitation, (d) Precipitation of driest month, (e) DEM, (f) Human population density, (g) Geomorphic type, (h) Land use type.

Based on the obtained response curves (Figure 6) for the Chinese giant salamander, the model logistic output (interpreted as the probability of Chinese giant salamander presence) during the Ming Dynasty, Qing Dynasty, and modern times shows an unimodal response pattern in the curves of bio3, minimum temperature of the coldest month (bio6), annual precipitation (bio12), precipitation of the driest month (bio14), DEM, and population density. As environmental factors increase, the probability of Chinese giant salamander presence increases, reaching a peak and then decreasing.

The ranges of isothermality (bio3), minimum temperature of the coldest month (bio6), annual precipitation (bio12), and precipitation of the driest month (bio14) values suitable for the survival of the Chinese giant salamander in the Chinese region (i.e., where the suitability probability > 0.32) show little difference between the Ming Dynasty, Qing Dynasty, and modern times. The variables with significant differences between historical and modern periods are the elevation range and geomorphic types (as shown in Figure 6). At that time, the suitable elevation range was 0–1300 m, while in modern times, it was 400–2400 m. In the Ming and Qing dynasties, the model suggests that the Chinese giant salamander was commonly found in plains, hills, and low mountain areas, while in modern times, the salamander is more likely to be found in low and high mountainous regions. In historical times, Chinese giant salamanders were frequently found in villages, croplands, and seminatural areas. This shift in distribution likely reflects changes in the habitats available to the species due to human activities and environmental changes rather than a change in habitat preference.

## 4 Discussion

### 4.1 Continued Impact of Historical Human Activities on the Distribution Range of the Chinese Giant Salamander

The results of this study indicate that the Chinese giant salamander was once widely distributed in the middle and lower reaches of the Yangtze River, but its habitat has gradually shifted westward and contracted from the Ming Dynasty to the present. While previous studies have documented significant declines in the species' population and habitat range (Turvey et al. 2019; Wang et al. 2004), our research brings a novel perspective by highlighting the sustained influence of human activities over centuries. Specifically, we reveal how historical shifts in human population density have long shaped the salamander's distribution. Turvey et al. (2018) emphasize that human‐mediated translocations and habitat fragmentation have disrupted biogeographic patterns, complicating modern conservation efforts. Similarly, Wang et al. (2004) identify habitat loss and overhunting since the 1950s as major threats to the species. Building on these findings, our study provides a broader temporal lens, showing that anthropogenic pressures from as far back as the Ming Dynasty have contributed to the species' westward habitat shift.

Our findings show an increase in population density and expansion of urban and village areas, which have been linked to habitat loss and fragmentation, driven by hunting, land conversion, and infrastructure development. These factors are recognized as primary contributors to the decline of many species, including the Chinese giant salamander (Burnham and Anderson 2004; McKee, Chambers, and Guseman 2013). Additionally, previous research has shown that areas with higher population densities tend to have fewer nature reserves, more agricultural land, and higher road densities, which exacerbate the threats to biodiversity (Liao et al. 2019). Thus, population growth and associated land use changes have been pivotal in driving the contraction of the Chinese giant salamander's distribution.

From 1650 to 1949, cultivated land area generally increased while forest coverage decreased. Over centuries, nearly half of the expanded cultivated land came from deforestation in China (Ge et al. 2008). Historical data further show that forest coverage in China decreased from 26% during the Ming Dynasty to 21% during the Qing Dynasty, and even fell to 12.5% in the modern era (Fan and Dong 2001). Although reforestation efforts have increased the total forest coverage to over 20% (Feng et al. 2023), the proportion of natural forests has gradually declined, accounting for only 30% of the total forest area by 2004 (Li 2004). This pattern reflects a continuing trend of habitat degradation as human activities intensified.

Additionally, climate change and human expansion not only impact the geographic distribution of the Chinese giant salamander but also reduce habitat quality. Urbanization not only alters river hydrology but also pollutes water sources (Price et al. 2011), potentially placing the species in unsuitable environments, as it prefers fast‐flowing, clean waters (Chen et al. 2018). Furthermore, the decline in groundwater levels and reduced relative humidity pose threats to its breeding habitats (Mammola et al. 2019).

Moreover, the mystique and cultural significance of the Chinese giant salamander have historically provided some protection, suggesting that cultural attitudes have played a role in conservation efforts (Cunningham et al. 2016; Wen 2018). However, in the early modern era, the shift in perception and increased demand for its meat and supposed medicinal properties have led to overexploitation, which continues to threaten its population despite legal protections (Wang et al. 2004; Zhao et al. 2018). These anthropogenic pressures have exacerbated the threats to the Chinese giant salamander, leading to its current precarious status.

Our study also highlights the complexity of predicting species distribution changes under climate change scenarios. While it is evident that *Andrias davidanus* is shifting its range to adapt to rising temperatures, the full impact of human‐induced land‐use changes complicates this dynamic. This underscores the need for integrated conservation strategies that address both climate change and human impacts on natural habitats. The survival of the Chinese giant salamander is intricately linked to our ability to mitigate these dual threats. Conservation efforts must prioritize habitat restoration, stringent enforcement of wildlife protection laws, and the promotion of cultural values that support the conservation of this unique species.

The unimodal relationship observed between the probability of species presence and human population density in our study might seem counterintuitive at first but can be explained by a combination of ecological and anthropogenic factors. At lower population densities, human activities such as agriculture and small‐scale settlements can enhance habitat suitability by creating a mosaic of diverse habitats, increasing the availability of food and shelter for *Andrias davidianus* (Marzluff 2001; McKinney 2002). This results in a positive correlation between population density and species presence. However, as population density increases beyond a certain threshold, the intensity of human activities such as urbanization and infrastructure development leads to habitat degradation and pollution, reducing habitat suitability and causing a decline in the probability of species presence (Blair 1996; Boakes et al. 2010). The unimodal relationship observed can be explained by the balance between moderate human disturbance, which fosters ecological niches, and excessive human impact, which leads to habitat degradation. At lower population densities, human activities such as small‐scale agriculture and settlements can create heterogeneous landscapes, enhancing habitat suitability by providing necessary resources, such as food and shelter, for species like *Andrias davidianus* (Huston 2005; McKinney 2008). However, as human population density increases beyond a certain threshold, these activities transition toward urbanization and large‐scale land conversion. This shift results in habitat fragmentation, pollution, and overall environmental degradation, rendering habitats increasingly unsuitable for the species (Foley et al. 2005; Grimm et al. 2008).

Furthermore, historical records are often biased toward more densely populated areas where human activity and documentation are more frequent. This bias can lead to an overrepresentation of species presence in these areas and an underestimation in less populated regions (Boakes et al. 2010; Graham et al. 2008). For example, in their study on geographical sampling bias, Reddy and Dávalos (2003) found that bird species in South America were significantly under‐recorded in less accessible regions, leading to an incomplete understanding of their distribution. Similarly, McKinney (2008) discussed how species richness near urban areas is often overestimated due to the high frequency of documentation, while species in remote or high‐elevation habitats tend to be under‐recorded. These biases should be considered when interpreting the distribution data for *Andrias davidianus* , as historical records may reflect not only actual habitat suitability but also the intensity of human observation. Therefore, the shift in the potential habitat of the Chinese giant salamander to higher elevations may represent a contraction rather than a true range shift. Our habitat simulation results further support this, indicating that the salamander's habitat loss is primarily concentrated in the lower‐elevation eastern regions, while higher‐elevation western habitats remain intact, suggesting a contraction of the salamander's distribution range rather than a westward migration.

### 4.2 Ecological Shifts and Conservation Implications for Chinese Giant Salamanders

The observed westward and northward shifts in the distribution of Chinese giant salamanders, as identified in our study, align with global patterns observed in other species facing similar environmental pressures (Chen et al. 2011). These shifts highlight the species' adaptation strategies in response to both climatic changes and increased human activity. The persistence of their climatic ecological niche since the Ming Dynasty suggests a notable resilience to climatic variations, but this stability is now challenged by accelerated human developments and more severe climate changes.

The transition to higher elevations in modern times reflects the elimination of salamander populations from lower elevations due to severe anthropogenic disturbances, leading to their remaining populations being restricted to less disturbed mountainous regions. This shift is crucial for the survival of the species, as their traditional distribution is increasingly encroached upon by urban expansion and agricultural intensification (Chen et al. 2018). The preference for less disturbed mountainous regions may reduce immediate threats but highlights a concerning trend toward habitat fragmentation, which can isolate populations and reduce genetic diversity (Alex Smith and Green 2005). While higher elevation areas may have supported salamander populations historically, under‐sampling in these less densely populated regions has likely led to an incomplete record of their historical presence. More recent surveys have provided a clearer understanding of their current distribution in these regions (Pyke and Ehrlich 2010; Reddy and Dávalos 2003).

Our research findings indicate that despite the persistence of its climatic ecological niche since the Ming Dynasty, the Chinese giant salamander is expected to continue shifting its distribution northward under various RCP scenarios. This observed stability in its ecological niche, wherein the species has adapted to climatic conditions for centuries, is being challenged by ongoing climate changes and human activities. Amphibians, such as *Andrias davidianus* , are particularly vulnerable due to their ectothermic nature, which ties their life cycles closely to external climatic conditions (Bartelt, Klaver, and Porter 2010). As winter temperatures rise, the salamander may emerge earlier from hibernation, increasing energy expenditure and reducing growth rates (Caruso et al. 2014). However, its reliance on fast‐flowing, clean aquatic habitats, which are often fragmented due to human infrastructure and land use, limits its ability to follow the shifting climatic zones necessary for its survival (Tingley et al. 2012). Thus, while the salamander has demonstrated resilience to historical climatic fluctuations, modern habitat fragmentation, and climate change pose significant threats, potentially leading to population bottlenecks or local extinctions in regions where suitable habitats become inaccessible (Moritz et al. 2008).

Given these challenges, conservation efforts must prioritize creating and maintaining ecological corridors that facilitate range shifts and connect isolated populations. For instance, the Yellowstone to Yukon Conservation Initiative (Y2Y) (Chester 2015), which spans over 3200 km, has proven successful in connecting habitats and enabling the migration of species such as wolves and grizzly bears. This initiative demonstrates the potential of large‐scale corridor projects to support species movement and genetic diversity. Similarly, the Pan‐European Ecological Network (PEEN) has played a key role in connecting fragmented habitats across Europe, facilitating the movement of species like the lynx and brown bear and thereby reducing the risk of population isolation (Bennett and Mulongoy 2006).

Moreover, integrating community‐based conservation programs is essential to enhance the effectiveness of these strategies, as local communities play a critical role in monitoring and protecting these habitats. Evidence from various community‐led conservation initiatives, such as the UNDP Equator Initiative projects, illustrates how partnerships between local stakeholders and conservation agencies have successfully strengthened both biodiversity conservation and local livelihoods (Berkes 2007). However, addressing potential challenges in engaging local communities, such as ensuring equitable participation and managing conflicting interests, is crucial for enhancing the practicality of these strategies. Engaging local stakeholders not only aids in direct conservation efforts but also helps mitigate anthropogenic pressures that contribute to habitat degradation.

## 5 Conclusion

This study utilized the MaxEnt model and ensemble models to reconstruct the historical distribution of the Chinese giant salamander, revealing the significant impacts of both climatic factors and human activities. Our analysis shows that precipitation and temperature have consistently dominated distribution patterns, while human activities have also played a crucial role. We observed a notable reduction in suitable habitats over time, with a 10% decrease from the Ming to the Qing Dynasty and a further 30% decrease into the modern era, particularly in the eastern plains. The loss of critical aquatic environments highlights the severe consequences of human encroachment and environmental degradation. Future projections indicate continued habitat decline and northward shifts under high carbon emission scenarios, posing a high risk of local extinctions due to the species' limited migratory capabilities.

To mitigate these risks, conservation efforts should prioritize habitat protection, environmental restoration, and stricter regulation of human activities affecting the species. Emphasizing sustainable practices and reducing carbon emissions are also crucial. Future research should refine predictive models by incorporating genetic data and remote sensing technologies to monitor habitat changes and assess species' resilience. Engaging local communities in conservation efforts will be vital for long‐term success.

## Author Contributions

**Siqing Li:** conceptualization (lead), data curation (equal), formal analysis (equal), methodology (lead), visualization (lead), writing – original draft (lead). **Wenyu Dai:** formal analysis (equal), methodology (equal), writing – review and editing (equal). **Zhenkang Wang:** data curation (equal), formal analysis (equal). **Zhaoning Wu:** data curation (equal), methodology (supporting), visualization (supporting), writing – original draft (supporting). **Jiechen Wang:** data curation (lead), funding acquisition (lead), project administration (lead), resources (lead), visualization (equal), writing – review and editing (lead).

## Conflicts of Interest

The authors declare no conflicts of interest.

## Supporting information


Data S1.

Data S2.

## Data Availability

We used open‐access data from GBIF (https://www.gbif.org/), WorldClim (http://www.worldclim.org/; https://doi.org/10.1002/ece3.3994), World Data Center for Renewable Resources and Environment (http://www.geodata.cn), and HYDE (History Database of the Global Environment) 3.2 database (https://doi.org/10.17026/dans‐25g‐gez3.; https://doi.org/10.5194/essd‐9‐927‐2017), Socioeconomic Data and Applications Center (SEDAC) (https://sedac.ciesin.columbia.edu/; https://doi.org/10.7927/H4Z60M4Z). The data of Chinese giant salamander in Qing Dynasty and Ming Dynasty used in this study were placed in the Data [Link], [Link].
