# Supplementary material for: Detecting Range Shrinking From Historical Amphibian Species Occurrences Under Influence of Human Impacts: A Case Study Using the Chinese Giant Salamander, Andrias davidianus
Source: Ecol Evol. 2024 Nov 19;14(11):e70595. doi: 10.1002/ece3.70595 (PMC11576131; doi:10.1002/ece3.70595)
Supplement: Supplementary file 1 — Data S1. [file ECE3-14-e70595-s001.docx]

Table S1. Occurrence records of Chinese giant salamander during Ming Dynasty.

| No. | Latitude | Longitude | Locality |
| --- | --- | --- | --- |
| 1 | 118.436 | 29.87112 | Ziyang Mountain in She County, Anhui Province |
| 2 | 118.7752 | 30.9908 | Xuanzhou County, Anhui Province |
| 3 | 115.3893 | 28.85936 | Zhanggong District, Ganzhou City, Jiangxi Province |
| 4 | 115.0497 | 25.92672 | Gan County District, Jiangxi Province |
| 5 | 114.9439 | 25.39302 | Xinfeng County, Jiangxi Province |
| 6 | 115.4109 | 25.04903 | Anyuan County, Jiangxi Province |
| 7 | 116.0218 | 26.40041 | Ningdu County, Jiangxi Province |
| 8 | 116.3468 | 26.31794 | Shicheng County, Jiangxi Province |
| 9 | 114.2794 | 35.74111 | Xinfeng County, Jiangxi Province |
| 10 | 114.2794 | 35.74111 | Ci County Fuyang River, Henan Province |
| 11 | 113.5689 | 33.43032 | Wuyang County, Henan Province |
| 12 | 113.1151 | 33.29579 | Fangcheng County, Henan Province |
| 13 | 114.1947 | 33.1359 | Nanru River, Henan Province |
| 14 | 113.3246 | 32.74006 | Biyang County, Henan Province |
| 15 | 112.2839 | 35.15331 | Jiyuan City Wangwu Mountain, Henan Province |
| 16 | 108.9718 | 30.30419 | Lichuan County, Enshi Prefecture, Hubei Province |
| 17 | 109.0691 | 29.70013 | Xianfeng County, Enshi Prefecture, Hubei Province |
| 18 | 110.0674 | 29.92517 | Hefeng County, Enshi Prefecture, Hubei Province |
| 19 | 109.4808 | 30.32598 | Enshi County, Enshi Prefecture, Hubei Province |
| 20 | 110.6029 | 25.70802 | Panlong Mountain, Guilin City, Guangxi Province |
| 21 | 102.8622 | 29.8048 | Rongjing County, Sichuan Province |
| 22 | 102.9196 | 30.14288 | Lushan County, Sichuan Province |
| 23 | 103.1166 | 30.06891 | Mingshan County, Sichuan Province |
| 24 | 109.2194 | 34.38548 | Linhe Jiangzhai Site, Lintong District, Shanxi Province |
| 25 | 109.5668 | 34.52361 | Zhushan, Linwei District, Weinan City, Shanxi Province |
| 26 | 110.3505 | 34.20319 | Between Luonan County and Huashan, Shanxi Province |
| 27 | 109.7943 | 33.93155 | Xiongershan, Shangzhou District, Shanxi Province |
| 28 | 110.3387 | 33.71259 | Danfeng County, Shanxi Province |
| 29 | 115.3893 | 28.85936 | Zhanggong District, Ganzhou City, Jiangxi Province |
| 30 | 115.0497 | 25.92672 | Gan County District, Jiangxi Province |
| 31 | 114.9439 | 25.39302 | Xinfeng County, Jiangxi Province |
| 32 | 115.4109 | 25.04903 | Anyuan County, Jiangxi Province |
| 33 | 116.0218 | 26.40041 | Ningdu County, Jiangxi Province |
| 34 | 116.3468 | 26.31794 | Shicheng County, Jiangxi Province |
| 35 | 107.2214 | 35.68182 | Zhenyuan County, Guizhou Province |
| 36 | 103.4009 | 29.8996 | Hongya County, Sichuan Province |

Table S2. Occurrence records of Chinese giant salamander during Qing Dynasty.

| No. | Latitude | Longitude | Locality |
| --- | --- | --- | --- |
| 1 | 119.7414 | 30.24935 | Lin'an County, Zhejiang Province |
| 2 | 119.4865 | 30.58156 | Wulong Pond in Tianmu Mountain, Zhejiang Province |
| 3 | 120.4972 | 27.66926 | Pingyang County, Zhejiang Province |
| 4 | 119.6335 | 27.9992 | Jingning She Autonomous County, Zhejiang Province |
| 5 | 118.4107 | 31.35656 | Wuhu County, Anhui Province |
| 6 | 118.4761 | 30.95148 | Nanling County, Anhui Province |
| 7 | 118.567 | 30.09654 | Jixi County, Anhui Province |
| 8 | 118.985 | 30.64432 | Ningguo, Anhui Province |
| 9 | 118.4333 | 30.722 | Jing County, Anhui Province |
| 10 | 119.1564 | 31.12211 | Langxi County, Anhui Province |
| 11 | 119.4432 | 30.84872 | Guangde County, Anhui Province |
| 12 | 115.3893 | 28.85936 | Jing'an County, Jiangxi Province |
| 13 | 118.2862 | 27.07426 | Wuning County, Jiangxi Province |
| 14 | 115.4303 | 28.44179 | Gao'an City, Jiangxi Province |
| 15 | 114.9931 | 28.25104 | Shanggao County, Jiangxi Province |
| 16 | 114.8118 | 28.39529 | Yifeng County, Jiangxi Province |
| 17 | 113.0214 | 34.51992 | Song Mountain, Henan Province |
| 18 | 112.1898 | 34.55199 | Yiyang County Yanliang River, Henan Province |
| 19 | 111.8759 | 34.78244 | Yima County, Henan Province |
| 20 | 111.314 | 34.11795 | Lushi County Xionger Mountain, Henan Province |
| 21 | 112.797 | 34.83341 | Yanshi County, Henan Province |
| 22 | 112.5381 | 34.16988 | Ruyang County, Henan Province |
| 23 | 112.1424 | 34.14455 | Song County, Henan Province |
| 24 | 114.7969 | 30.85419 | Xinzhou District, Wuhan, Hubei Province |
| 25 | 110.2491 | 32.22688 | Zhushan County, Hubei Province |
| 26 | 110.7488 | 32.09905 | Fang County, Hubei Province |
| 27 | 113.1957 | 31.39258 | Jingshan County Tiansheng Weir, Hubei Province |
| 28 | 114.598 | 31.30516 | Hong'an County, Hubei Province |
| 29 | 115.6421 | 29.84844 | Wuxue County, Hubei Province |
| 30 | 111.8078 | 30.8379 | Dangyang County, Hubei Province |
| 31 | 112.3176 | 30.07155 | Jiangling County, Hubei Province |
| 32 | 112.281 | 30.27104 | Jinzhou county, Hubei Province |
| 33 | 111.1882 | 30.48811 | Changyang Tujia Autonomous County, Hubei Province |
| 34 | 111.0695 | 30.16466 | Wufeng Tujia Autonomous County, Hubei Province |
| 35 | 110.9965 | 30.84341 | Zigui County, Hubei Province |
| 36 | 111.4657 | 30.42128 | Yidu County, Hubei Province |
| 37 | 111.6345 | 31.08147 | Yuan'an County, Hubei Province |
| 38 | 110.7469 | 25.70407 | Xing'an County, Hubei Province |
| 39 | 109.7294 | 30.60318 | Jianshi County, Hubei Province |
| 40 | 109.4566 | 29.52509 | Laifeng County, Hubei Province |
| 41 | 108.9689 | 30.2997 | Lichuan City, Hubei Province |
| 42 | 111.1386 | 29.45461 | Cili County, Hunan Province |
| 43 | 111.5431 | 28.98253 | Taoyuan County, Hunan Province |
| 44 | 111.4054 | 29.59841 | Shimen County, Hunan Province |
| 45 | 112.1097 | 26.80084 | Qidong County, Hunan Province |
| 46 | 112.6699 | 26.95214 | Hengyang City, Hunan Province |
| 47 | 112.4487 | 26.56744 | Changning County, Hunan Province |
| 48 | 112.8867 | 26.42703 | Leiyang County, Hunan Province |
| 49 | 112.6681 | 26.95317 | Hengyang County, Hunan Province |
| 50 | 112.898 | 27.25141 | Hengshan County, Hunan Province |
| 51 | 112.9739 | 27.09013 | Hengdong County, Hunan Province |
| 52 | 112.411 | 26.42025 | Changning County, Hunan Province |
| 53 | 113.2492 | 26.73955 | Anren County Yongle River, Hunan Province |
| 54 | 113.9608 | 26.04942 | Guidong County, Hunan Province |
| 55 | 111.6321 | 25.53305 | Daoxian County, Hunan Province |
| 56 | 111.6171 | 25.18507 | Jianghua Yao Autonomous County, Hunan Province |
| 57 | 110.1687 | 26.58842 | Suining County, Hunan Province |
| 58 | 111.2733 | 26.99859 | Shaoyang County, Hunan Province |
| 59 | 109.7186 | 26.59166 | Jingzhou Miao Autonomous County, Hunan Province |
| 60 | 110.4142 | 28.45191 | Yuanling County, Hunan Province |
| 61 | 110.5866 | 27.92214 | Xupu County, Hunan Province |
| 62 | 110.2532 | 27.98658 | Chenxi County, Hunan Province |
| 63 | 111.3293 | 27.71629 | Xinhua County, Hunan Province |
| 64 | 110.2352 | 28.27584 | Luxi County, Hunan Province |
| 65 | 109.4654 | 29.46865 | Longshan County, Hunan Province |
| 66 | 112.3116 | 24.73417 | Liannan Yao Autonomous County, Guangdong Province |
| 67 | 113.6133 | 24.82742 | Shaoguan City, Guangdong Province |
| 68 | 110.6866 | 25.62487 | Xing'an, Guilin, Guangxi Province |
| 69 | 110.6763 | 26.0735 | Ziyuan County, Guangxi Province |
| 70 | 110.3613 | 25.39936 | Lingchuan County, Guangxi Province |
| 71 | 107.7691 | 29.88344 | Fengdu County, Chongqing Province |
| 72 | 103.9138 | 30.8112 | Pixian County, Sichuan Province |
| 73 | 103.7869 | 30.6361 | Chongzhou County, Sichuan Province |
| 74 | 103.6196 | 30.99079 | Dujiangyan County, Sichuan Province |
| 75 | 103.6196 | 30.99079 | Dujiangyan County, Sichuan Province |
| 76 | 103.583 | 29.72478 | Jiajiang County, Sichuan Province |
| 77 | 103.5525 | 28.84174 | Mabian County, Sichuan Province |
| 78 | 104.7259 | 28.44585 | Gong County, Sichuan Province |
| 79 | 108.0507 | 32.08204 | Wanyuan County, Sichuan Province |
| 80 | 102.9196 | 30.14288 | Lushan County, Sichuan Province |
| 81 | 102.0492 | 31.93935 | Dadu River, Aba Prefecture, Sichuan Province |
| 82 | 102.1051 | 31.49038 | Jinchuan County, Sichuan Province |
| 83 | 102.3924 | 31.00221 | Xiaojin County, Sichuan Province |
| 84 | 101.9028 | 30.87584 | Danba County, Sichuan Province |
| 85 | 106.9859 | 27.07397 | Kaiyang County, Guizhou Province |
| 86 | 106.6123 | 26.84332 | Xiuwen County, Guizhou Province |
| 87 | 106.7424 | 27.08773 | Xifeng County, Guizhou Province |
| 88 | 106.4167 | 27.79315 | Renhuai County, Guizhou Province |
| 89 | 107.0385 | 27.73958 | Zunyi County, Guizhou Province |
| 90 | 106.8486 | 28.13437 | Tongzi County, Guizhou Province |
| 91 | 107.9181 | 28.55233 | Wuchuan Gelao and Miao Autonomous County, Guizhou Province |
| 92 | 107.4801 | 27.75143 | Meitan County, Guizhou Province |
| 93 | 106.2803 | 26.40808 | Pingba County, Guizhou Province |
| 94 | 108.9258 | 27.26259 | Yuping Dong Autonomous County, Guizhou Province |
| 95 | 107.9834 | 26.56998 | Kaili County, Guizhou Province |
| 96 | 107.2532 | 26.58549 | Guiding County, Guizhou Province |
| 97 | 106.7522 | 25.4371 | Luodian County, Guizhou Province |
| 98 | 106.4624 | 26.01911 | Changshun County, Guizhou Province |
| 99 | 108.7384 | 34.33452 | Xianyang County, Shanxi Province |
| 100 | 107.7878 | 33.00006 | Xixiang County, Shanxi Province |
| 101 | 107.9141 | 32.55501 | Zhenba County, Shanxi Province |
| 102 | 107.5818 | 33.23853 | Yang County, Shanxi Province |
| 103 | 111.096 | 39.05756 | Fugu County, Shanxi Province |

Table S3. Occurrence records of Chinese giant salamander during modern period from Wen’s work (2018).

| No. | Latitude | Longitude | Locality |
| --- | --- | --- | --- |
| 1 | 112.0328 | 35.37515 | Majiahe, Shanxi Province |
| 2 | 112.0133 | 35.34769 | Longwang Mountain, Zhejiang Province |
| 3 | 119.4424 | 30.41965 | Xianliang Town, Zhejiang Province |
| 4 | 119.2392 | 27.65322 | Tianzhu Mountain, Anhui Porvince |
| 5 | 116.4667 | 30.74191 | Tiantangzhai, Anhui Province |
| 6 | 115.7853 | 31.14113 | Shuanghe Village, Anhui Province |
| 7 | 116.6019 | 31.11609 | Foziling Town, Anhui Province |
| 8 | 116.2618 | 31.3662 | Ketian Village, Xianyu Town, Shitai, Anhui Province |
| 9 | 117.3443 | 30.07307 | Tongmu Village, Wuyishan National Park, Fujian Province |
| 10 | 117.6868 | 27.75319 | Hua'an County, Fujian Province |
| 11 | 117.5489 | 25.05453 | Wanshan Village, Bailu Town, Lushan City, Jiangxi Province |
| 12 | 116.016 | 29.48509 | Wugong Mountain, Pingxiang, Jiangxi Province |
| 13 | 114.1359 | 27.49532 | Jiuling Mountain, Jiangxi Province |
| 14 | 115.279 | 28.97754 | Laoguan River, Tanghe Township, Lushi County, Henan Province |
| 15 | 111.1629 | 33.88668 | Baishui River, Maotang Township, Xichuan Public Security Bureau, Henan Province |
| 16 | 111.4119 | 33.23849 | Xinye County, Nanyang, Henan Province |
| 17 | 112.3596 | 32.58312 | Xieqiao Village, Lijiazhai Town, Xinyang, Henan Province |
| 18 | 114.1049 | 31.89748 | Jinlian Cave Forest Area, Shiyan City, Hubei Province |
| 19 | 109.8407 | 32.45606 | Yuhe Village, Maliao Gorge, Baokang County, Hubei Province |
| 20 | 111.4266 | 31.46326 | Wudao Gorge Scenic Area, Hubei Province |
| 21 | 111.2343 | 31.73011 | Dalao Ridge, Yichang, Hubei Province |
| 22 | 110.9576 | 31.04704 | Xingdou Mountain, Enshi, Hubei Province |
| 23 | 109.1249 | 30.03612 | Bamao Creek, Hubei Province |
| 24 | 110.0551 | 29.66104 | Siduping, Hubei Province |
| 25 | 110.4481 | 28.93461 | Wudao Water Town, Hubei Province |
| 26 | 109.9366 | 29.71644 | Suoxi, Wulingyuan, Hunan Province |
| 27 | 110.6081 | 29.37017 | Sanguansi, Hunan Province |
| 28 | 110.6627 | 29.43181 | Lishu Village, Huajiang Town, Hunan Province |
| 29 | 111.7173 | 29.90864 | Heng Mountain, Nanyue, Hunan Province |
| 30 | 112.7221 | 27.27567 | Micang Mountain, Sichuan Province |
| 31 | 106.8726 | 32.72038 | Wenxi River, Tingzi Lake, Jiange County, Sichuan Province |
| 32 | 105.7562 | 31.93616 | Guangwu Mountain, Nanjiang County, Sichuan Province |
| 33 | 106.8072 | 32.69495 | Gouxi River, Sichuan Province |
| 34 | 106.0901 | 31.55127 | Juewu Village, Shilu Town, Hejiang County, Sichuan Province |
| 35 | 105.8421 | 28.75167 | Caojia Tuojiang, Jiang'an, Yibin, Sichuan Province |
| 36 | 105.0961 | 28.73048 | Xingwen County, Sichuan Province |
| 37 | 104.9252 | 28.20527 | Taoxi Valley, Bailixia Nature Reserve, Sichuan Province |
| 38 | 115.4194 | 39.72366 | Zongren Valley, Sichuan Province |
| 39 | 107.1028 | 30.86242 | Taiji Village, Jinsha County, Guizhou Province |
| 40 | 106.2271 | 27.45646 | Liannan Yao Autonomous County, Guangdong Province |
| 41 | 112.3136 | 24.7391 | Sijian Mountain, Rongshui Miao Autonomous County, Guangxi Province |
| 42 | 108.9474 | 25.1703 | Lingkou Chinese Giant Salamander Nature Reserve, Guangxi Province |
| 43 | 110.7213 | 33.22203 | Majiahe, Shanxi Province |

Table S4. Occurrence records of Chinese giant salamander during modern period from GBIF.

| No. | Latitude | Longitude | gbifID |
| --- | --- | --- | --- |
| 1 | 110.581744 | 33.738167 | 4076221529 |
| 2 | 119.599454 | 26.738711 | 3986189991 |
| 3 | 110.55 | 29.34 | 3862907301 |
| 4 | 110.548175 | 29.262811 | 3499446977 |
| 5 | 114.171712 | 28.428151 | 3337414192 |
| 6 | 103.360159 | 30.323446 | 3058825649 |
