# Supplementary material for: Detecting Range Shrinking From Historical Amphibian Species Occurrences Under Influence of Human Impacts: A Case Study Using the Chinese Giant Salamander, Andrias davidianus
Source: Ecol Evol. 2024 Nov 19;14(11):e70595. doi: 10.1002/ece3.70595 (PMC11576131; doi:10.1002/ece3.70595)
Supplement: Supplementary file 2 — Data S2. [file ECE3-14-e70595-s002.docx]

Supplementary Material

1. **Maxent Modeling**
   Maxent Modeling: The Maxent 3.3.3 software was used, applying the Maxent algorithm as outlined by Phillips et al. (2006). The ENMeval R package (Muscarella et al., 2014) was utilized to optimize Maxent parameter settings. Maxent models were computed using various parameter combinations to balance the trade-off between model fit and overfitting. The ENMeval was configured to test regularization multiplier values at 1 and 2 steps, along with feature classes including Linear (L), Quadratic (Q), Product (P), Threshold (T), and Hinge (H), with combinations such as L, H, LQ, LQP, LQH, LQHP, and LQHPT. In the ENMeval workflow, 100,000 random points were generated to serve as background points. Model selection was performed using the small sample size corrected Akaike Information Criterion (AICc) (Burnham & Anderson, 2004).
2. **MaxEnt Model Optimization and Performance**

This section provides details on the optimization process and performance metrics for the MaxEnt model, which was used as a comparative method to support the robustness of the ensemble models presented in the main manuscript.

**Table S1.** Performance Comparison of the MaxEnt Model with Default and Optimized Parameters for the Chinese Giant Salamander in China across the Ming Dynasty, Qing Dynasty, and Modern Periods. "FC" represents Feature Class combinations in the model: Linear (L), Quadratic (Q), Product (P), Threshold (T), and Hinge (H). "RM" is the Regularization Multiplier, which adjusts model complexity. "ΔAICc" shows the difference in corrected Akaike Information Criterion between default and optimized settings. "OR_10" is the omission rate at the 10th percentile training presence threshold, reflecting the proportion of known occurrences misclassified as absent.

| Period | Setting | FC | RM | ΔAICc | OR_10 |
| --- | --- | --- | --- | --- | --- |
| Ming Dynasty | Default | LQHPT | 1 | 41.58 | 0.22 |
|  | Optimized | LQHPT | 2 | 0 | 0.05 |
| Qing Dynasty | Default | LQHPT | 1 | 40.56 | 0.21 |
|  | Optimized | LQHPT | 2 | 0 | 0.04 |
| Modern Period | Default | LQHPT | 1 | 70.05 | 0.22 |
|  | Optimized | LQHP | 2 | 0 | 0.04 |

**Modeling Process:**

The MaxEnt model was optimized using the ENMeval R package (Muscarella et al., 2014). A range of feature classes (FC) and regularization multiplier (RM) settings were tested to balance model fit and prevent overfitting. The optimized models showed a significant improvement in performance compared to the default settings, as indicated by lower ΔAICc values and reduced OR_10 rates (Table S1), reflecting enhanced accuracy and predictive reliability.

**Performance Metrics:**

The Area Under the Curve (AUC) for the MaxEnt models ranged from 0.921 to 0.941 across the Ming, Qing, and modern periods, indicating robust model performance well above random chance expectations (Table S2).

The True Skill Statistic (TSS) values for the MaxEnt models varied from 0.70 to 0.78, further confirming the model's predictive strength.

**Table S2.** Performance Metrics (AUC and TSS) of MaxEnt Models for Chinese Giant Salamander Habitat Suitability Across Different Historical Periods

| **Period** | **AUC** | **TSS** |
| --- | --- | --- |
| MING | 0.921 | 0.70 |
| QING | 0.934 | 0.78 |
| MODERN | 0.941 | 0.74 |

**Correlation with Ensemble Models:**

The correlation between the habitat suitability maps predicted by the MaxEnt model and those generated by the ensemble model were very high: 0.948 for the Ming period, 0.952 for the Qing period, and 0.938 for the Modern period. This strong agreement between the two modeling approaches supports the reliability of the ensemble models used in the main analysis.

1. **Species Distribution Shifts Using MaxEnt Model**

This section provides detailed results from the MaxEnt model for the historical periods analyzed in the main study. These results support the findings presented using the ensemble models and provide an additional comparative perspective.

**TABLE S3.** Species Distribution Shifts for the Chinese Giant Salamander Across Different Historical Periods using MaxEnt

| Time Period | Suitable Area (km²) | Reduction Ratio (%) | Average Elevation (m) | Centroid (Longitude, Latitude) |
| --- | --- | --- | --- | --- |
| Ming Dynasty | 1.41 × 10¹² | - | 441.91 | 112.53°E, 30.40°N |
| Qing Dynasty | 1.28 × 10¹² | 9.32 | 457.57 | 111.80°E, 29.63°N |
| Modern | 9.30 × 10¹¹ | 27.65 | 864.40 | 110.52°E, 29.28°N |

The MaxEnt model indicated similar trends to the ensemble model, with a significant reduction in suitable habitat area and an upward shift in the centroid and average elevation over time.


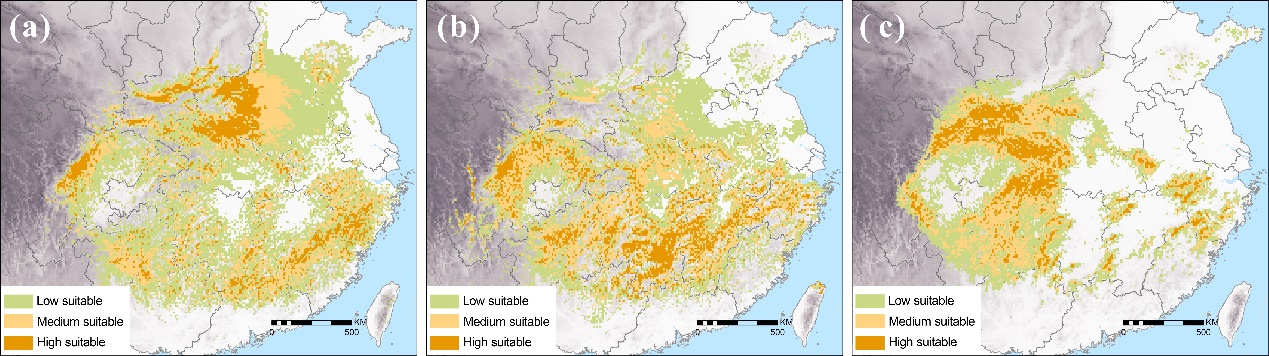


Figure S1. Potential suitable habitat of the Chinese giant salamander in China during (a) the Ming Dynasty, (b) the Qing Dynasty, and (c) the Modern period based on MaxEnt model results. The background is a Digital Elevation Model (DEM), with elevation depicted in blue. Colors indicate habitat suitability: low (green), medium (orange), and high (dark orange).
